# Circulating Acylcarnitines Associated with Hypertrophic Cardiomyopathy Severity: an Exploratory Cross-Sectional Study in *MYBPC3* Founder Variant Carriers

**DOI:** 10.1007/s12265-023-10398-2

**Published:** 2023-06-06

**Authors:** Mark Jansen, A. F. Schmidt, J. J. M. Jans, I. Christiaans, S. N. van der Crabben, Y. M. Hoedemaekers, D. Dooijes, J. D. H. Jongbloed, L. G. Boven, R. H. Lekanne Deprez, A. A. M. Wilde, J. van der Velden, R. A. de Boer, J. P. van Tintelen, F. W. Asselbergs, A. F. Baas

**Affiliations:** 1 Department of Genetics, University Medical Center Utrecht, Utrecht University, Utrecht, the Netherlands; 2 Department of Cardiology, University Medical Center Utrecht, Utrecht University, Internal Mail No HTx Secr. (E03.511), Postbus 85500, 3508 GA Utrecht, the Netherlands; 3 Netherlands Heart Institute, Utrecht, the Netherlands; 4 http://guardheart.ernnet.eu; 5 Institute of Cardiovascular Science, Faculty of Population Health Sciences, University College London, London, UK; 6 Department of Cardiology, Amsterdam UMC Location University of Amsterdam, Amsterdam, the Netherlands; 7 Amsterdam Cardiovascular Sciences, Heart Failure and Arrhythmias, Amsterdam, the Netherlands; 8 Department of Genetics, University of Groningen, University Medical Center Groningen, Groningen, the Netherlands; 9 Department of Human Genetics, Amsterdam UMC, University of Amsterdam, Amsterdam, the Netherlands; 10 Department of Clinical Genetics, Radboud University Medical Center, Nijmegen, the Netherlands; 11 Department of Physiology, Amsterdam UMC, Location Vrije Universiteit Amsterdam, Amsterdam, the Netherlands; 12 Department of Cardiology, University of Groningen, University Medical Center Groningen, Groningen, the Netherlands; 13 Department of Cardiology, Erasmus Medical Center, Rotterdam, the Netherlands; 14 Health Data Research UK and Institute of Health Informatics, University College London, London, UK

**Keywords:** Hypertrophic Cardiomyopathy, *MYBPC3*, Biomarker, Acylcarnitine, Metabolism

## Abstract

Hypertrophic cardiomyopathy (HCM) is a relatively common genetic heart disease characterised by myocardial hypertrophy. HCM can cause outflow tract obstruction, sudden cardiac death and heart failure, but severity is highly variable. In this exploratory cross-sectional study, circulating acylcarnitines were assessed as potential biomarkers in 124 *MYBPC3* founder variant carriers (59 with severe HCM, 26 with mild HCM and 39 phenotype-negative [G + P-]). Elastic net logistic regression identified eight acylcarnitines associated with HCM severity. C3, C4, C6-DC, C8:1, C16, C18 and C18:2 were significantly increased in severe HCM compared to G + P-, and C3, C6-DC, C8:1 and C18 in mild HCM compared to G + P-. In multivariable linear regression, C6-DC and C8:1 correlated to log-transformed maximum wall thickness (coefficient 5.01, *p* = 0.005 and coefficient 0.803, *p* = 0.007, respectively), and C6-DC to log-transformed ejection fraction (coefficient -2.50, *p* = 0.004). Acylcarnitines seem promising biomarkers for HCM severity, however prospective studies are required to determine their prognostic value.

**Graphical abstract:**

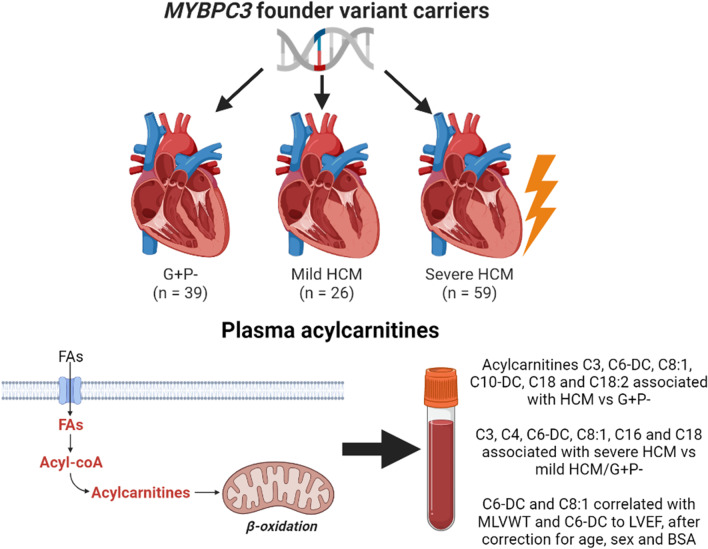

**Supplementary Information:**

The online version contains supplementary material available at 10.1007/s12265-023-10398-2.

## Introduction

Hypertrophic cardiomyopathy (HCM) is the most common Mendelian cardiac disease and an important cause of sudden cardiac death [1–3]. While many patients exhibit little to no symptoms, HCM may present with left ventricular outflow tract obstruction (LVOTO) requiring septal reduction therapy (SRT), and a small proportion of patients experiences malignant ventricular arrhythmia (MVA) and/or debilitating heart failure (HF) [4, 5]. Inheritance is typically autosomal dominant with incomplete penetrance [6]. Pathogenic variants are predominantly found in genes encoding cardiac sarcomere proteins, with (likely) pathogenic genetic variants identified in about 50% of patients in historical, well-characterised cohorts [7, 8]. Based on contemporary, more heterogeneous cohorts, the yield of genetic testing in HCM is estimated at 33% [9]. The most frequently affected gene is *MYBPC3*, which encodes cardiac myosin-binding protein C, a key regulator of cardiomyocyte contractility [10]. Founder variants in *MYBPC3*, including c.2373dupG, c.2827C > T, c.2864_2865delCT or c.3776delA, are identified in 20–35% of HCM cases in the Netherlands [11]. These variants lead to nonsense mediated mRNA decay of the mutant allele, resulting in haploinsufficiency [12].

Impaired energy metabolism has been proposed as a key pathomechanism in HCM [13]. In particular, studies have shown impaired myocardial fatty acid metabolism, including changes in myocardial acylcarnitines [14–16]. Acylcarnitines are conjugations of acyl groups, primarily derived from fatty acids, with carnitine, which transport the acyl groups from the cytosol into mitochondria for energy production [17]. Changes in plasma acylcarnitines reflect the dysregulation of fatty acid metabolism in tissue, especially the heart [18, 19]. Consistently, untargeted (non-quantitative) metabolomics studies suggested myocardial changes in acylcarnitines are mirrored in circulating acylcarnitines in HCM [20–22]. However, these findings still require confirmation using quantitative methods to assess whether circulating acylcarnitines can function as minimally-invasive biomarkers for HCM severity.

In this exploratory study, we quantitatively measured plasma acylcarnitines and employed a feature selection algorithm to assess possible associations between plasma acylcarnitines and HCM severity, as well as echocardiographic surrogates, in 124 carriers of *MYBPC3* founder variants across the phenotypic spectrum.

## Methods

### Subject Inclusion

This study consisted of a cross-sectional analysis of the *BIO FOr CARe* study (Identification of biomarkers of hypertrophic cardiomyopathy development and progression in Dutch *MYBPC3* founder variant carriers), which included carriers of the c.2373dupG, c.2827C > T, c.2864_2865delCT and c.3776delA *MYBPC3* founder variants aged ≥ 18 years from three Dutch University Medical Centres (Utrecht, Groningen and Amsterdam). The design of this study and patient inclusion was previously published [23]. Subjects underwent prospective blood collection for biomarker assessment. Patients with prior heart transplantation were excluded. The study was approved by the institutional review board of the University Medical Centre Utrecht and all subjects provided informed consent.

### Acylcarnitine Profiling

Acylcarnitine profiles were measured in plasma at the metabolic diagnostics laboratory of the University Medical Centre Utrecht. Plasma was derived from heparinised blood samples, collected under non-fasting, resting conditions and centrifuged for 10 min at 2000 g, and subsequently stored at -80 °C until analysis [23].

Samples were butylated and evaporated (using nitrogen) at 40 °C, after which concentrations of 42 acylcarnitines were analysed using tandem liquid chromatography-mass spectrometry (LC–MS; Acquity UPLC system [serial numbers of components: E12UPAB979A, F12UPA794M, C12UPM597G and E12UPO328M] & Xevo TQ MS [serial number VBA 760], Waters, Milford, Massachusetts), as previously published [24]. Calibration curves were used for quantification. Missing acylcarnitine values were assumed to reflect concentrations below the detection limit of LC–MS, and were replaced by half the minimum observed value of each acylcarnitine.

Acylcarnitines are referred to by their acyl-chain carbon chain length (C0-C18), the presence and number of unsaturated bonds (indicated as counts after colons [:]), and the presence of hydroxyl- (-OH) or dicarboxyl (-DC) groups.

### Study Outcomes & Definitions

Subjects were classified into severe HCM, mild HCM and genotype-positive phenotype-negative (G + P-). Severe HCM phenotype was defined as a composite endpoint composed of a documented maximum left ventricular wall thickness (MLVWT) ≥ 20 mm, LVOTO necessitating SRT, HF (clinical diagnosis of congestive HF, requiring treatment with loop diuretics, or left ventricular ejection fraction [LVEF] < 50%) or MVA (sustained ventricular tachycardia, ventricular fibrillation, appropriate implantable cardioverter-defibrillator intervention, resuscitated cardiac arrest, or sudden cardiac death). Sustained ventricular tachycardia was defined as > 30 s, with haemodynamic instability or requiring earlier termination. Sudden cardiac death was defined as death due to documented ventricular arrhythmia or within 1 h of symptom onset, in absence of other identified causes. Subjects fulfilling HCM criteria but no criteria for severe HCM were classified as mild HCM. HCM was defined as a MLVWT ≥ 13 mm, not explained by abnormal loading conditions [1]. Subjects that did not fulfil HCM criteria were classified as G + P-.

Additionally, MLVWT, indexed left atrial volume (LAVi) and LVEF were assessed as continuous variables.

### Statistical Analysis

Baseline characteristics were presented as counts with percentages for dichotomous and categorical variables and medians with interquartile ranges for continuous variables, and compared using Chi-square tests (or Fisher-Freeman Halton tests when observed counts were < 10) and Kruskal–Wallis tests, respectively. Correlations between acylcarnitines were assessed using Spearman’s correlation coefficient and visualised using a heatmap.

To identify acylcarnitines associated with HCM severity, feature selection was performed using elastic net logistic regression with the “glmnet” and “caret” packages [25, 26]. Age and sex were additionally included as candidate predictors. Continuous variables were mean-centred and scaled to one standard deviation. Model hyperparameters were selected using 10-times repeated fivefold cross validation. Boxplots were used to visualise associations of acylcarnitines with HCM severity. Associations were tested using Mann Whitney U and Kruskal–Wallis tests.

Acylcarnitines were correlated to echocardiographic parameters using linear regression. Multivariable linear regression was used to correct for age, sex and body surface area (BSA). Logarithmic transformations were explored to improve model fit, specifically exploring potential deviations from a standard normal distribution, linearity or homoscedasticity. Missing values in BSA and echocardiographic parameters were imputed using multiple imputation by chained equations [27]. Based on Von Hippol’s two-stage calculation, 125 imputations were used [28].

Sensitivity analyses were performed stratified by HF and SRT, using the composite endpoint for severe HCM without the MLVWT ≥ 20 mm endpoint, using a complete-case analysis, removing participants with missing data instead of imputing these values, and excluding subjects with SRT from linear regression analysis for MLVWT.

In this exploratory analysis, *p*-values < 0.05 were considered statistically significant. All analyses were performed in R version 4.1.2 (R Development Core Team, 2020).

## Results

A total of 124 subjects were included. Severe HCM was identified in 59 subjects (47.6%). Specifically, 47 subjects had a documented MLVWT ≥ 20 mm, 10 underwent SRT, 11 experienced MVA and 27 experienced HF (congestive HF in 14, LVEF < 50% in 16). The overlap between the constituent endpoints is shown in Supplemental Figure 1. Mild HCM was identified in 26 subjects (21.0%). The remaining 39 subjects were G + P- (31.5%). Subject characteristics are presented in Table 1.

**Table 1:** Subject characteristics

	G + P-	Mild HCM	Severe HCM	*P*-value
	(*n* = 39)	(*n* = 26)	(*n* = 59)	
Age (years)	47.3 [32.9, 56.1]	66.2 [52.8, 69.8]	56.8 [47.0, 68.6]	**0.001**
Male sex	12 (30.8)	12 (46.2)	40 (67.8)	**0.001**
Body surface area (m^2^)	1.9 [1.8, 2.0]	1.9 [1.7, 2.0]	2.0 [1.9, 2.2]	**0.008**
Family history of SCD	11 (28.2)	16 (61.5)	22 (38.6)	**0.028**
Unexplained non-vasovagal syncope	1 (2.6)	3 (11.5)	11 (19.0)	**0.042**
NYHA class III/IV	0 (0.0)	0 (0.0)	8 (20.0)	**0.002**
Non-sustained VT	6 (28.6)	13 (61.9)	35 (64.8)	**0.017**
MLVWT (mm)	10 [9, 11]	14 [13,16]	20 [17, 23]	** < 0.001**
LVEF (%)	60 [60, 64]	60 [58, 65]	60 [50, 61]	**0.004**
LVOT gradient (mmHg)	5 [3, 6]	5 [4, 7]	5 [4, 12]	0.22
LAVi (ml/m^2^)	26 [22, 29]	36 [28, 42]	48 [36, 63]	** < 0.001**
Atrial fibrillation	2 (5.1)	3 (11.5)	27 (46.6)	** < 0.001**
Concomitant hypertension	8 (20.5)	8 (30.8)	18 (31.0)	0.51

Data are shown as counts (%), means (standard deviation) or medians [interquartile range]. *P*-values were determined across all three groups; *p*-values < 0.05 are shown in bold. *G* + *P-* Genotype-positive phenotype-negative; *HCM* Hypertrophic cardiomyopathy; *LAVi* Indexed left atrial volume; *LVEF* Left ventricular ejection fraction; *LVOT* Left ventricular outflow tract; *MLVWT* Maximum left ventricular wall thickness; *NYHA* New York Heart Association; *SCD* Sudden cardiac death; *VT* Ventricular tachycardia

Acylcarnitine concentrations are provided in Supplemental Table 1. Correlations between acylcarnitines are shown in Supplemental Figure 2. Correlations ranged from -0.14 to 0.95. The median absolute correlation was 0.26 (interquartile range 0.15–0.39). The median of the strongest absolute correlation per acylcarnitine pair was 0.64 (interquartile range 0.50–0.71), with absolute correlations > 0.8 in six pairs of acylcarnitines (C8-C10, C10-C12:1, C12-C12:1, C12-C14, C12-C14:1, C12:1-C14:1).

### Acylcarnitines Associated with HCM Severity

Acylcarnitines C3, C4, C6-DC, C8:1, C16, C18 and sex associated with severe HCM compared to mild HCM and G + P-. Acylcarnitines C3, C6-DC, C8:1, C10-DC, C18, C18:2, sex and age associated with HCM (both mild and severe) compared to G + P-. Odds ratios are provided in Table 2.

**Table 2:** Acylcarnitine selection

	Severe HCM vs G + P-/mild HCM	Mild/severe HCM vs G + P-
Male sex	2.124	1.827
Age	Not selected	1.371
C3	1.001	1.224
C4	1.075	Not selected
C6-DC	1.291	1.222
C8:1	1.095	1.311
C10-DC	Not selected	1.029
C16	1.105	Not selected
C18	1.028	1.108
C18:2	Not selected	1.045

Odds ratios for the acylcarnitines selected by the elastic net logistic regression models for severe HCM versus G + P-/mild HCM and mild/severe HCM versus G + P-. Continuous variables were scaled and centred. Acylcarnitines C0, C2, C3-DC, C4-DC, C4:3-OH, C5, C5-DC, C5-OH, C5:1, C6, C6-OH, C6:1, C7, C8, C8-DC, C10, C10:1, C10:2, C12, C12-DC, C12-OH, C12:1, C14, C14-OH, C14:1, C14:2, C16-DC, C16-OH, C16:1, C16:1-OH, C18-OH, C18:1, C18:1-DC, C18:1-OH, and C18:2-OH were not selected by either model. *G* + *P-* genotype-positive phenotype negative; *HCM* Hypertrophic cardiomyopathy; *vs* Versus

**Fig. 1:**
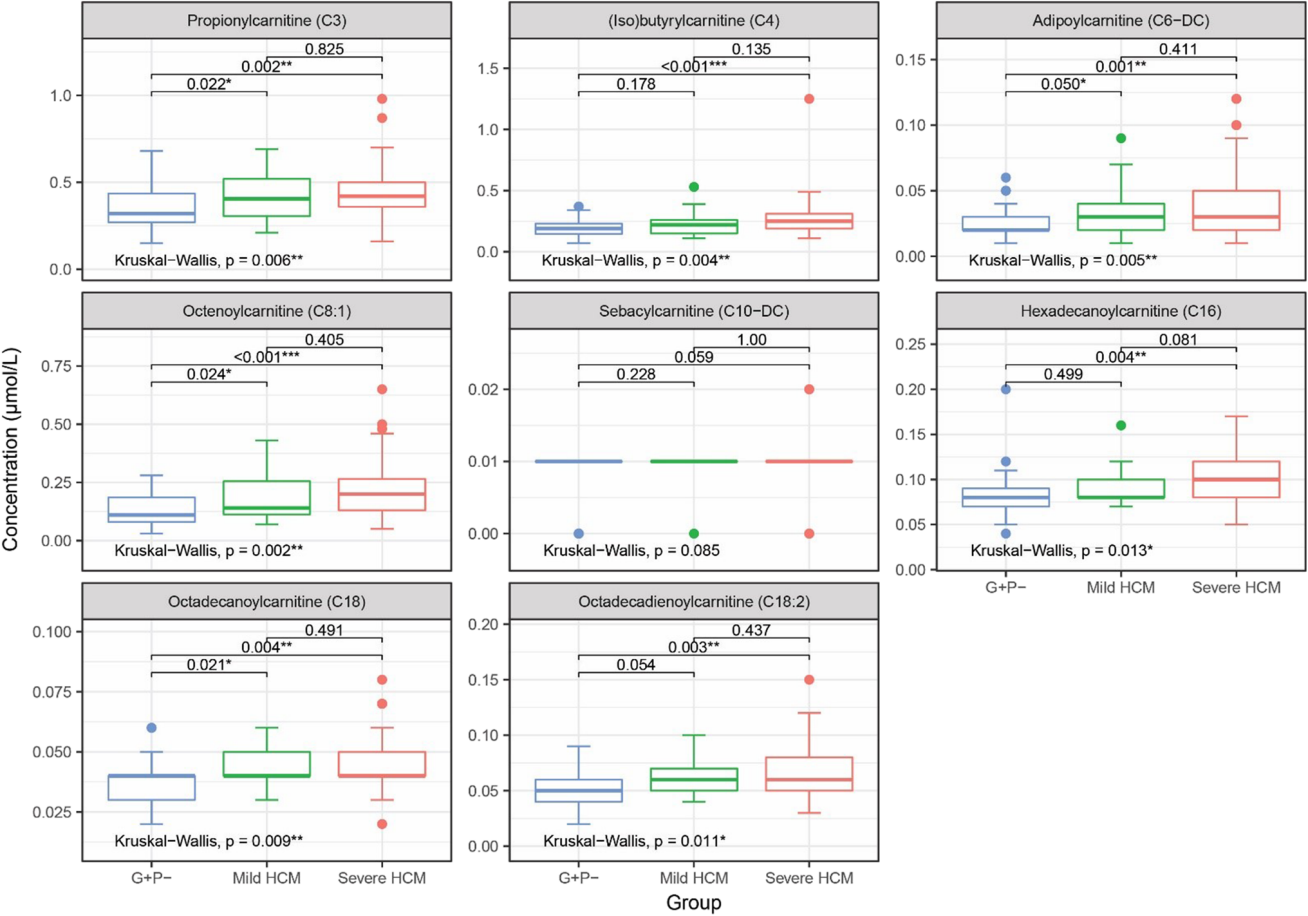
Boxplots of selected acylcarnitines. Boxplots showing concentrations of the acylcarnitines selected by elastic net logistic regression, with pairwise and overall *p*-values from Mann–Whitney U and Kruskal–Wallis tests, respectively. *p < 0.05, **p < 0.01, ***p < 0.001. G + P-, genotype-positive phenotype-negative; HCM, hypertrophic cardiomyopathy

Boxplots for the selected acylcarnitines are shown in Fig. 1. Acylcarnitines C3, C4, C6-DC, C8:1, C16, C18 and C18:2 were significantly increased in subjects with severe HCM compared to G + P-. Additionally, C3, C6-DC, C8:1 and C18 were significantly increased in subjects with mild HCM compared to G + P-.

### Correlations with Clinical Variables

Results from linear regression analyses are shown in Table 3. In univariable analysis, acylcarnitines C3, C4, C6-DC, C8:1 and C18:2 were significantly correlated with MLVWT. C6-DC additionally correlated with LAVi and LVEF. After correction for age, sex and BSA in multivariable analyses, C6-DC and C8:1 remained significantly correlated with MLVWT, and C6-DC with LVEF.

**Table 3:** Correlations of acylcarnitines with clinical variables

Univariable	log(MLVWT)		log(LAVi)		log(LVEF)	
	Coefficient	*P*-value	Coefficient	*P*-value	Coefficient	*P*-value
log(C3)	0.251	**0.010**	0.219	0.161	-0.036	0.509
log(C4)	0.208	**0.015**	0.263	0.463	-0.067	0.117
C6-DC	3.61	**0.024**	5.88	**0.024**	-2.35	**0.002**
C8:1	0.823	**0.007**	0.497	0.313	-0.224	0.140
C10-DC	19.3	0.086	12.5	0.445	-9.11	0.094
log(C16)	0.155	0.215	0.209	0.272	-0.129	**0.043**
log(C18)	0.125	0.316	0.141	0.513	-0.035	0.592
log(C18:2)	0.199	**0.043**	0.097	0.515	-0.111	**0.024**
Multivariable	log(MLVWT)		log(LAVi)		log(LVEF)	
	Adjusted coefficient	*P*-value	Adjusted coefficient	*P*-value	Adjusted coefficient	*P*-value
log(C3)	0.124	0.223	0.033	0.835	0.029	0.582
log(C4)	0.129	0.141	0.125	0.366	-0.024	0.575
C6-DC	5.01	**0.005**	5.13	0.054	-2.50	**0.004**
C8:1	0.803	**0.007**	0.178	0.697	-0.158	0.293
C10-DC	12.4	0.257	2.79	0.862	-5.99	0.256
log(C16)	0.060	0.629	0.040	0.842	-0.083	0.181
log(C18)	-0.009	0.946	-0.118	0.621	0.043	0.497
log(C18:2)	0.096	0.359	-0.077	0.633	-0.064	0.201

Results from univariable and multivariable linear regression (adjusted for age, sex and body surface area). *P*-values < 0.05 are shown in bold. *LAVi* Indexed left atrial volume; *LVEF* Left ventricular ejection fraction; *LVOT* Left ventricular outflow gradient; *MLVWT* Maximum wall thickness

### Sensitivity Analyses

Subject characteristics stratified by HF are shown in Supplemental Table 2, and analyses stratified by HF are shown in Supplemental Table 3. Acylcarnitines C3, C4 and C6-DC remained associated with severe HCM (versus mild HCM/G + P-) in the non-HF stratum, and C3, C6-DC, C8:1, C10-DC, C18 and C18:2 all remained associated with HCM (mild and severe together versus G + P-) in the non-HF stratum.

Subject characteristics stratified by SRT are shown in Supplemental Table 4, and analyses stratified by SRT are shown in Supplemental Table 5. Acylcarnitines C3, C4, C6-DC, C16 and C18 remained associated with severe HCM in the non-SRT stratum, and C3, C6-DC, C8:1, C10-DC, C18 and C18:2 all remained associated with HCM in the non-SRT stratum.

Subject characteristics stratified by severe HCM without the MLVWT ≥ 20 mm endpoint are shown in Supplemental Table 6. Results of elastic net analysis are shown in Supplemental Table 7, with sex and acylcarnitines C6-DC, C8:1 and C16 remaining associated with the outcome.

As shown in Supplemental Table 8, point estimates were similarly directed in linear regression analyses using complete-case analysis. In multivariable analyses, C8:1 remained significantly correlated to MLVWT (*p* = 0.023) and C6-DC to LVEF (*p* = 0.003). Likewise, point estimates for MLVWT remained similar after exclusion of subjects with SRT, as shown in Supplemental Table 9. In multivariable analysis, C6-DC remained significantly correlated to MLVWT (*p* = 0.004).

## Discussion

In order to explore potential biomarkers for HCM development and/or progression, we determined plasma acylcarnitine profiles in 124 carriers of *MYBPC3* founder variants, including 59 severe HCM patients, 26 with mild HCM and 39 G + P- individuals. We identified profiles encompassing eight acylcarnitines that associated with HCM severity. Seven of these acylcarnitines (C3, C4, C6-DC, C8:1, C16, C18 and C18:2) were significantly increased in severe HCM compared to G + P, and four (C3, C6-DC, C8:1 and C18) were additionally increased in mild HCM compared to G + P-. After correction for age, sex and BSA, C6-DC was significantly correlated with MLVWT and LVEF and C8:1 with MLVWT.

### Fatty Acid Oxidation and Metabolic Treatment in HCM

Impaired energy metabolism has been proposed as a key pathomechanism in HCM [13]. HCM-causing genetic variants can lead to reductions in the energy-conserving super relaxed myosin state, which induces changes in cardiac metabolism [29, 30]. Particularly, cardiomyocytes shift away from fatty acid oxidation to utilise other energy sources [14–16].

Currently, two fatty acid metabolism modulators are being studied as treatment of HCM. The METAL-HCM phase 2 trial assessed perhexiline, a mitochondrial carnitine palmitoyltransferase-1 inhibitor, in symptomatic HCM patients, showing energetic and modest functional improvement [31]. However, further studies were delayed due to concerns regarding systemic toxicity and a phase 2 trial in patients with HCM and HF with preserved ejection fraction (NCT02862600) was terminated early due to lack of efficacy. RESOLVE-HCM, a phase 2 trial assessing the effects of perhexiline on MLVWT, is currently recruiting [32]. Trimetazidine, a mitochondrial long-chain 3‑ketoacyl coenzyme A thiolase inhibitor, was associated with a decline in functional parameters in a phase 2 trial on symptomatic patients with non-obstructive HCM [33]. The ENERGY trial, assessing whether trimetazidine may restore cardiac efficiency in G + P-, is currently underway [34].

Additionally, the cardiac myosin inhibitor mavacamten reduced LVOTO, symptoms and guideline-eligibility for SRT in obstructive HCM in two phase 3 trials, and was well-tolerated in a phase 2 trial in symptomatic non-obstructive HCM [35–37]. Another myosin inhibitor, aficamten, was shown to reduce LVOTO in patients with obstructive HCM a phase 2 trial [38]. These drugs likely ameliorate metabolic dysregulation in HCM by promoting the super-relaxed state of myosin and thereby reducing energetic demand, as treatment with mavacamten restored cellular oxygen consumption rates in induced pluripotent stem cell-derived cardiomyocytes carrying HCM-causing *MYH7* missense variants, and improved the number of dysregulated genes encoding mitochondrial proteins in mouse models of HCM [30, 39, 40].

### Myocardial and Circulating Acylcarnitines in HCM

Three multi-omics studies comparing tissue obtained from HCM patients undergoing septal myectomy to non-failing donor hearts confirmed changes in fatty acid oxidation [14–16]. Myocardial acylcarnitines were studied in two of these. Acylcarnitines transport acyl groups, primarily derived from fatty acids, from the cytosol into mitochondria for beta-oxidation [15]. Consistent with the changes in fatty acid oxidation, almost all of the determined acylcarnitines were significantly decreased [15, 16].

Several untargeted metabolomics studies suggested that the differences in myocardial acylcarnitines were mirrored in circulating acylcarnitines in HCM patients. Shimada et al. identified various acylcarnitines among the most important metabolites in plasma that discriminated exercise response between age-, sex- and body mass index-matched patients with HCM, left ventricular hypertrophy secondary to hypertension and other cardiovascular diseases [20]. Schuldt et al. found serum C5-DC among the most important metabolites in discriminating patients with LVOTO from unmatched asymptomatic carriers of HCM-associated genetic variants, with higher C5-DC in patients with LVOTO [21]. In our previous study, acylcarnitines C8:1, C16:2 and C20 were identified among the top metabolites in plasma to discriminate between severe HCM patients and age- and sex-matched patients with mild HCM and G + P-, with higher C8:1 and C20 and lower C16:2 in severely affected patients [22]. However, these studies used case–control designs and non-quantitative methods, therefore still requiring confirmation in studies with more robust designs using quantitative methods.

Nakamura et al. quantified acylcarnitines C0 and C2 in serum of HCM patients, showing that C0 was significantly increased and C2 significantly decreased compared to subjects with left ventricular hypertrophy secondary to hypertension and healthy controls [41, 42]. In HCM patients, C0 correlated to myocardial fatty acid metabolism assessed by [^123^I]-β-Methyl iodophenyl-pentadecanoic acid-imaging, which was significantly reduced compared to either control group.

In the present study, we performed a more comprehensive quantitative assessment of acylcarnitines using LC–MS, in a larger, genetically homogeneous cohort of HCM patients including G + P- relatives. This revealed several additional acylcarnitines associated with HCM severity. Consistent with the studies in myocardium, this included acylcarnitines of various acyl-chain lengths. The mechanisms connecting decreased myocardial levels of acylcarnitines to increased plasma levels remain unclear, however similarly directed changes in myocardial and plasma acylcarnitines have been described in dilated cardiomyopathy [43, 44].

The acylcarnitines identified in this study retained their associations with HCM after stratification for HF and SRT. However, the associations of C8:1, C16 and C18 with severe HCM were no longer observed after stratification for HF, suggesting that these associations were driven by HF. Additionally, we explored correlations with echocardiographic parameters for HCM, identifying correlations of C6-DC with MLVWT and LVEF and C8:1 with MLVWT independent of age, sex and BSA. Based on our data, acylcarnitines appear to be promising biomarkers for HCM severity.

### Study Limitations

Despite this being the largest study to assess circulating acylcarnitines in HCM, including G + P-, thus far, sample size was still insufficient to correct for additional potential confounders, including diabetes mellitus, renal function and medication usage. Additionally, this study included a relatively large number of subjects with end-stage HCM, characterised by HF, and subjects with previous SRT, which may limit generalisability. Previous positron emission tomography suggested attenuated myocardial energy demands after alcohol septal ablation, however effects of SRT on acylcarnitines have not yet been studied [45, 46]. In our study, most associations of acylcarnitines with HCM and many of the associations with severe HCM remained after stratification for HF and SRT, as well as after removing the MLVWT endpoint from the composite endpoint for severe HCM, suggesting robust associations with HCM severity.

The cross-sectional design of this study limits inference. Multiple testing may have resulted in type I error. Large prospective cohort studies are required to confirm our findings and assess the prognostic value of acylcarnitines in predicting specific clinical effects, on top of known predictors and other potentially predictive circulating biomarkers, such as natriuretic peptides, troponins, high-sensitivity C-reactive protein and uric acid [47].

Furthermore, our study cannot exclude extracardiac production as the source of the perturbed acylcarnitines, as we did not acquire tissue samples or invasive measurements. However, the heart was previously identified as the main contributor to plasma concentrations of acylcarnitines, particularly medium- and long-chain acylcarnitines (C6-C20), and six out of the eight acylcarnitines indicated by our study were previously shown to be significantly different in myocardium of HCM patients compared to healthy donor hearts [15, 17, 19]. Additionally, samples were obtained under non-fasting conditions, while a fasted state is likely more appropriate to assess myocardial mitochondrial dysfunction [17]. However, biomarkers should ideally remain predictive regardless of sampling conditions, and our data suggest acylcarnitines are associated to HCM severity when obtained in non-fasting conditions.

## Conclusion

The identification of HCM biomarkers is crucial to improve individualised risk prediction and thereby treatment of patients with this clinically heterogeneous disease. In this study of 124 carriers of *MYBPC3* founder variants across the phenotypic spectrum of HCM, profiles of eight circulating acylcarnitines were associated with HCM severity. Seven acylcarnitines (C3, C4, C6-DC, C8:1, C16, C18 and C18:2) were significantly increased in severe HCM compared to G + P, and four (C3, C6-DC, C8:1 and C18) were additionally increased in mild HCM compared to G + P-. C6-DC was significantly correlated with MLVWT and LVEF and C8:1 with MLVWT, after correction for age and sex. Acylcarnitines appear to be promising biomarkers for HCM severity, however further studies are required to assess their prognostic value.

### Supplementary Information

Below is the link to the electronic supplementary material.

Supplementary file1 (DOCX 758 kb)

## Data Availability

Data are available upon request from the authors.

## Abbreviations

G + P-: Genotype-positive, phenotype-negative
HCM: Hypertrophic cardiomyopathy
HF: Heart failure
LAVi: Indexed left atrial volume
LC-MS: Liquid chromatography-mass spectrometry
LVEF: Left ventricular ejection fraction
LVOTO: Left ventricular outflow tract obstruction
MLVWT: Maximum left ventricular wall thickness
MVA: Malignant ventricular arrhythmia
SRT: Septal reduction therapy
