# Supplementary material for: Circulating Acylcarnitines Associated with Hypertrophic Cardiomyopathy Severity: an Exploratory Cross-Sectional Study in MYBPC3 Founder Variant Carriers
Source: J Cardiovasc Transl Res. 2023 Jun 6;16(6):1267–75. doi: 10.1007/s12265-023-10398-2 (PMC10721678; doi:10.1007/s12265-023-10398-2)
Supplement: Supplementary file 1 — Supplementary file1 (DOCX 758 kb) [file 12265_2023_10398_MOESM1_ESM.docx]

**Circulating acylcarnitines associated with hypertrophic cardiomyopathy severity: an exploratory cross-sectional study in carriers of *MYBPC3* founder variants carriers**

Supplemental material

M Jansen et al. (2023)

Contents

[Supplemental Table 1. Acylcarnitine concentrations 2](#_Toc131688849)

[Supplemental Table 2. Subject characteristics – heart failure 4](#_Toc131688850)

[Supplemental Table 3. Sensitivity analysis – heart failure 5](#_Toc131688851)

[Supplemental Table 4. Subject characteristics – septal reduction therapy 6](#_Toc131688852)

[Supplemental Table 5. Sensitivity analysis – septal reduction therapy 7](#_Toc131688853)

[Supplemental Table 6. Subject characteristics – severe HCM without MLVWT ≥20 mm endpoint 8](#_Toc131688854)

[Supplemental Table 7. Sensitivity analysis – severe HCM without MLVWT ≥20 mm endpoint 9](#_Toc131688855)

[Supplemental Table 8. Sensitivity analysis linear regression – complete case analysis 10](#_Toc131688856)

[Supplemental Table 9. Sensitivity analysis linear regression – subjects without septal reduction therapy 11](#_Toc131688857)

[Supplemental Figure 1. Overlap between constituent endpoints 12](#_Toc131688858)

[Supplemental Figure 2. Correlations between acylcarnitines 13](#_Toc131688859)

## Supplemental Table 1. Acylcarnitine concentrations

| **Acylcarnitine** | **Median (IQR)** | **Mean ± SD** | **Range** |
| --- | --- | --- | --- |
| C0 | 42.83 (37.53-50.43) | 43.87 ± 10.24 | 15.5-70.44 |
| C2 | 3.96 (3.35-4.84) | 4.23 ± 1.32 | 1.19-8.23 |
| C3 | 0.40 (0.30-0.49) | 0.41 ± 0.14 | 0.15-0.98 |
| C3-DC | 0.04 (0.03-0.05) | 0.04 ± 0.02 | 0.01-0.12 |
| C4 | 0.22 (0.17-0.28) | 0.24 ± 0.13 | 0.07-1.25 |
| C4-DC | 0.04 (0.03-0.04) | 0.04 ± 0.01 | 0.00-0.08 |
| C4:3-OH | 0.02 (0.01-0.02) | 0.02 ± 0.01 | 0.00-0.06 |
| C5 | 0.14 (0.11-0.17) | 0.14 ± 0.05 | 0.04-0.28 |
| C5-DC | 0.04 (0.03-0.06) | 0.05 ± 0.02 | 0.02-0.12 |
| C5-OH | 0.01 (0.01-0.01) | 0.01 ± 0.01 | 0.00-0.03 |
| C5:1 | 0.01 (0.01-0.01) | 0.01 ± 0.01 | 0.00-0.05 |
| C6 | 0.03 (0.02-0.04) | 0.04 ± 0.02 | 0.01-0.18 |
| C6-DC | 0.03 (0.02-0.04) | 0.04 ± 0.02 | 0.01-0.12 |
| C6-OH | 0.01 (0.01-0.01) | 0.01 ± 0.01 | 0.00-0.03 |
| C6:1 | 0.00 (0.00-0.00) | 0.00 ± 0.00 | 0.00-0.01 |
| C7 | 0.00 (0.00-0.00) | 0.00 ± 0.00 | 0.00-0.02 |
| C8 | 0.09 (0.07-0.12) | 0.11 ± 0.10 | 0.03-1.03 |
| C8-DC | 0.01 (0.00-0.01) | 0.01 ± 0.01 | 0.00-0.04 |
| C8:1 | 0.16 (0.10-0.24) | 0.18 ± 0.11 | 0.03-0.65 |
| C10 | 0.15 (0.11-0.19) | 0.18 ± 0.13 | 0.05-1.10 |
| C10-DC | 0.01 (0.01-0.01) | 0.01 ± 0.00 | 0.00-0.02 |
| C10:1 | 0.10 (0.07-0.12) | 0.10 ± 0.05 | 0.03-0.30 |
| C10:2 | 0.01 (0.01-0.02) | 0.01 ± 0.01 | 0.00-0.08 |
| C12 | 0.04 (0.03-0.06) | 0.05 ± 0.03 | 0.01-0.21 |
| C12-DC | 0.01 (0.01-0.01) | 0.01 ± 0.00 | 0.00-0.01 |
| C12-OH | 0.01 (0.00-0.01) | 0.01 ± 0.01 | 0.00-0.02 |
| C12:1 | 0.04 (0.03-0.06) | 0.05 ± 0.03 | 0.01-0.22 |
| C14 | 0.02 (0.02-0.03) | 0.03 ± 0.01 | 0.01-0.09 |
| C14-OH | 0.01 (0.01-0.01) | 0.01 ± 0.00 | 0.00-0.02 |
| C14:1 | 0.05 (0.04-0.07) | 0.06 ± 0.04 | 0.01-0.30 |
| C14:2 | 0.03 (0.03-0.04) | 0.04 ± 0.02 | 0.01-0.13 |
| C16 | 0.09 (0.08-0.11) | 0.09 ± 0.03 | 0.04-0.20 |
| C16-DC | 0.00 (0.00-0.00) | 0.00 ± 0.00 | 0.00-0.01 |
| C16-OH | 0.00 (0.00-0.01) | 0.00 ± 0.01 | 0.00-0.01 |
| C16:1 | 0.02 (0.02-0.03) | 0.02 ± 0.01 | 0.01-0.11 |
| C16:1-OH | 0.01 (0.00-0.01) | 0.01 ± 0.01 | 0.00-0.02 |
| C18 | 0.04 (0.03-0.05) | 0.04 ± 0.01 | 0.02-0.08 |
| C18-OH | 0.00 (0.00-0.00) | 0.00 ± 0.00 | 0.00-0.01 |
| C18:1 | 0.13 (0.11-0.15) | 0.14 ± 0.05 | 0.06-0.40 |
| C18:1-DC | 0.01 (0.01-0.01) | 0.01 ± 0.00 | 0.00-0.03 |
| C18:1-OH | 0.00 (0.00-0.00) | 0.00 ± 0.00 | 0.00-0.01 |
| C18:2 | 0.06 (0.05-0.07) | 0.06 ± 0.02 | 0.02-0.15 |
| C18:2-OH | 0.00 (0.00-0.00) | 0.00 ± 0.00 | 0.00-0.01 |

Acylcarnitine concentrations in µmol/L. IQR, interquartile range; SD, standard deviation.

## Supplemental Table 2. Subject characteristics – heart failure

|  | **Severe HCM without heart failure** | **Severe HCM**  **with heart failure** | **P-value** |
| --- | --- | --- | --- |
|  | **(n = 32)** | **(n = 27)** |  |
| Age (years) | 53.2 [38.9, 59.8] | 65.0 [54.4, 72.0] | **0.008** |
| Male sex | 24 (75.0) | 16 (59.3) | 0.31 |
| Body surface area (m^2^) | 2.1 [2.0, 2.2] | 1.9 [1.8, 2.1] | **0.030** |
| Family history of SCD | 11 (35.5) | 11 (42.3) | 0.78 |
| Unexplained non-vasovagal syncope | 10 (31.2) | 1 (3.8) | **0.016** |
| NYHA class III/IV | 2 (9.1) | 6 (33.3) | 0.11 |
| Non-sustained VT | 16 (53.3) | 19 (79.2) | 0.084 |
| MLVWT (mm) | 21 [18, 23] | 17 [14, 21] | **0.007** |
| LVEF (%) | 60 [58, 65] | 50 [45, 60] | **<0.001** |
| LVOT gradient (mmHg) | 8 [4, 36] | 5 [3, 7] | **0.030** |
| LAVi (ml/m^2^) | 40 [34, 50] | 60 [49, 70] | **0.015** |
| Atrial fibrillation | 8 (25.8) | 19 (70.4) | **0.001** |
| Concomitant hypertension | 8 (25.8) | 10 (37.0) | 0.40 |

Data are shown as counts (%), means (standard deviation) or medians [interquartile range]. P-values <0.05 are shown in bold. HCM, hypertrophic cardiomyopathy; LAVi, indexed left atrial volume; LVEF, left ventricular ejection fraction; LVOT, left ventricular outflow tract; MLVWT, maximum left ventricular wall thickness; NYHA, New York Heart Association; SCD, sudden cardiac death; VT, ventricular tachycardia.

## Supplemental Table 3. Sensitivity analysis – heart failure

|  | **Severe HCM vs mild HCM/G+P-** | | **Mild/severe HCM vs G+P-** | |
| --- | --- | --- | --- | --- |
|  | **Stratum: Without heart failure** | **Stratum: With heart failure** | **Stratum: Without heart failure** | **Stratum:**  **With heart failure** |
| Male sex | Not selected | 16.97 | Not selected | 21.43 |
| Female sex | 0.2529 | 13.57 | 0.3756 | 18.05 |
| Age | - | - | 1.564 | Not selected |
| C3 | 1.345 | Not selected | 1.859 | Not selected |
| C4 | 1.125 | Not selected | - | - |
| C6-DC | 1.278 | Not selected | 1.906 | Not selected |
| C8:1 | Not selected | Not selected | 1.826 | Not selected |
| C10-DC | - | - | 1.278 | Not selected |
| C16 | Not selected | Not selected | - | - |
| C18 | Not selected | Not selected | 1.169 | Not selected |
| C18:2 | - | - | 1.220 | Not selected |

Odds ratios for the acylcarnitines selected by the elastic net logistic regression models stratified by heart failure. Continuous variables were scaled and centred. G+P-, genotype-positive phenotype negative; HCM, hypertrophic cardiomyopathy; vs, versus.

## Supplemental Table 4. Subject characteristics – septal reduction therapy

|  | **Severe HCM without septal reduction therapy** | **Severe HCM**  **with septal reduction therapy** | **P-value** |
| --- | --- | --- | --- |
|  | **(n = 49)** | **(n = 10)** |  |
| Age (years) | 57.7 [51.6, 71.0] | 43.9 [36.7, 53.5] | **0.029** |
| Male sex | 33 (67.3) | 7 (70.0) | 1.0 |
| Body surface area (m^2^) | 2.0 [1.9, 2.2] | 2.1 [2.0, 2.5] | 0.34 |
| Family history of SCD | 17 (36.2) | 5 (50.0) | 0.48 |
| Unexplained non-vasovagal syncope | 7 (14.6) | 4 (40.0) | 0.083 |
| NYHA class III/IV | 4 (11.8) | 4 (66.7) | **0.010** |
| Non-sustained VT | 31 (67.4) | 4 (50.0) | 0.43 |
| MLVWT (mm) | 20 [17, 22] | 23 [18, 29] | 0.11 |
| LVEF (%) | 60 [49, 62] | 59 [50, 60] | 0.69 |
| LVOT gradient (mmHg) | 5 [4, 11] | 6 [4, 22] | 0.68 |
| LAVi (ml/m^2^) | 49 [36, 63] | 48 [41, 54] | 0.86 |
| Atrial fibrillation | 23 (46.9) | 4 (44.4) | 1.0 |
| Concomitant hypertension | 16 (33.3) | 2 (20.0) | 0.71 |

Data are shown as counts (%), means (standard deviation) or medians [interquartile range]. P-values <0.05 are shown in bold. HCM, hypertrophic cardiomyopathy; LAVi, indexed left atrial volume; LVEF, left ventricular ejection fraction; LVOT, left ventricular outflow tract; MLVWT, maximum left ventricular wall thickness; NYHA, New York Heart Association; SCD, sudden cardiac death; VT, ventricular tachycardia.

## Supplemental Table 5. Sensitivity analysis – septal reduction therapy

|  | **Severe HCM vs mild HCM/G+P-** | | **Mild/severe HCM vs G+P-** | |
| --- | --- | --- | --- | --- |
|  | **Stratum: Without SRT** | **Stratum:**  **With SRT** | **Stratum: Without SRT** | **Stratum:**  **With SRT** |
| Male sex | Not selected | 14.52 | Not selected | 1.059 |
| Female sex | 0.2899 | 6.484 | 0.3126 | Not selected |
| Age | - | - | 1.961 | Not selected |
| C3 | 1.023 | Not selected | 1.590 | Not selected |
| C4 | 1.403 | Not selected | - | - |
| C6-DC | 1.800 | Not selected | 2.076 | Not selected |
| C8:1 |  | 1.255 | 1.364 | Not selected |
| C10-DC | - | - | 1.179 | 8.461E+06 |
| C16 | 1.074 | Not selected | - | - |
| C18 | 1.248 | Not selected | 1.266 | Not selected |
| C18:2 | - | - | 1.238 | Not selected |

Odds ratios for the acylcarnitines selected by the elastic net logistic regression models stratified by SRT. Continuous variables were scaled and centred. G+P-, genotype-positive phenotype negative; HCM, hypertrophic cardiomyopathy; SRT, septal reduction therapy; vs, versus.

## Supplemental Table 6. Subject characteristics – severe HCM without MLVWT ≥20 mm endpoint

|  | **Severe HCM without MLVWT ≥20 mm** | **Severe HCM solely due to MLVWT ≥20 mm** | **P-value** |
| --- | --- | --- | --- |
|  | **(n = 37)** | **(n = 22)** |  |
| Age (years) | 57.7 [47.0, 71.0] | 56.2 [46.5, 60.7] | 0.45 |
| Male sex | 23 (62.2) | 17 (77.3) | 0.36 |
| Body surface area (m^2^) | 2.0 [1.9, 2.1] | 2.2 [2.0, 2.2] | 0.054 |
| Family history of SCD | 16 (44.4) | 6 (28.6) | 0.27 |
| Unexplained non-vasovagal syncope | 7 (19.4) | 4 (18.2) | 1.0 |
| NYHA class III/IV | 8 (33.3) | 0 (0.0) | **0.013** |
| Non-sustained VT | 25 (73.5) | 10 (50.0) | 0.139 |
| MLVWT (mm) | 18 [15, 23] | 21 [20, 22] | 0.11 |
| LVEF (%) | 51 [48, 60] | 60 [59, 66] | **0.001** |
| LVOT gradient (mmHg) | 5 [4, 10] | 8 [6, 21] | 0.10 |
| LAVi (ml/m^2^) | 52 [42, 64] | 37 [34, 47] | **0.035** |
| Atrial fibrillation | 23 (63.9) | 4 (18.2) | **0.001** |
| Concomitant hypertension | 13 (35.1) | 5 (23.8) | 0.56 |

Data are shown as counts (%), means (standard deviation) or medians [interquartile range]. P-values <0.05 are shown in bold. HCM, hypertrophic cardiomyopathy; LAVi, indexed left atrial volume; LVEF, left ventricular ejection fraction; LVOT, left ventricular outflow tract; MLVWT, maximum left ventricular wall thickness; NYHA, New York Heart Association; SCD, sudden cardiac death; VT, ventricular tachycardia.

## Supplemental Table 7. Sensitivity analysis – severe HCM without MLVWT ≥20 mm endpoint

|  | **Severe HCM vs**  **G+P-/mild HCM** |
| --- | --- |
| Male sex | 1.376 |
| C3 | Not selected |
| C4 | Not selected |
| C6-DC | 1.199 |
| C8:1 | 1.301 |
| C16 | 1.496 |
| C18 | Not selected |

Odds ratios for the acylcarnitines selected by the elastic net logistic regression for severe HCM without the MLVWT ≥20 mm endpoint versus G+P-/mild HCM. Continuous variables were scaled and centred. G+P-, genotype-positive phenotype negative; HCM, hypertrophic cardiomyopathy; vs, versus.

## Supplemental Table 8. Sensitivity analysis linear regression – complete case analysis

| **Univariable** | **log(MLVWT)** | | **log(LAVi)** | | **log(LVEF)** | |
| --- | --- | --- | --- | --- | --- | --- |
|  | **Coefficient** | **P-value** | **Coefficient** | **P-value** | **Coefficient** | **P-value** |
| log(C3) | 0.332 | **0.001** | 0.363 | **0.010** | -0.022 | 0.636 |
| log(C4) | 0.254 | **0.002** | 0.350 | **0.005** | -0.060 | 0.118 |
| C6-DC | 3.75 | **0.018** | 6.08 | **0.003** | -2.39 | **0.001** |
| C8:1 | 0.912 | **0.002** | 0.347 | 0.399 | -0.231 | 0.103 |
| C10-DC | 18.8 | 0.072 | 16.2 | 0.289 | -8.82 | 0.079 |
| log(C16) | 0.168 | 0.176 | 0.420 | **0.018** | -0.119 | **0.038** |
| log(C18) | 0.170 | 0.161 | 0.257 | 0.140 | -0.027 | 0.638 |
| log(C18:2) | 0.205 | **0.035** | 0.112 | 0.397 | -0.104 | **0.019** |
|  |  | |  | |  | |
| **Multivariable** | **log(MLVWT)** | | **log(LAVi)** | | **log(LVEF)** | |
|  | **Adjusted coefficient** | **P-value** | **Adjusted coefficient** | **P-value** | **Adjusted coefficient** | **P-value** |
| log(C3) | 0.212 | 0.085 | 0.094 | 0.535 | 0.030 | 0.645 |
| log(C4) | 0.189 | 0.068 | 0.235 | 0.077 | -0.098 | 0.082 |
| C6-DC | 3.56 | 0.070 | 3.83 | 0.085 | -2.88 | **0.003** |
| C8:1 | 0.742 | **0.023** | 0.065 | 0.867 | -0.173 | 0.338 |
| C10-DC | 10.3 | 0.422 | 4.55 | 0.758 | -10.9 | 0.116 |
| log(C16) | 0.262 | 0.085 | 0.233 | 0.249 | -0.141 | 0.082 |
| log(C18) | 0.141 | 0.323 | -0.082 | 0.672 | 0.059 | 0.437 |
| log(C18:2) | 0.184 | 0.139 | -0.057 | 0.697 | -0.080 | 0.226 |

Results from univariable and multivariable linear regression (adjusted for age, sex and body surface area). MLVWT, maximum wall thickness; LVOT, left ventricular outflow gradient; LAVi, indexed left atrial volume; LVEF, left ventricular ejection fraction.

## Supplemental Table 9. Sensitivity analysis linear regression – subjects without septal reduction therapy

| **Univariable** | **log(MLVWT)** | |
| --- | --- | --- |
|  | **Coefficient** | **P-value** |
| log(C3) | 0.237 | **0.021** |
| log(C4) | 0.220 | **0.007** |
| C6-DC | 4.28 | **0.007** |
| C8:1 | 0.675 | **0.031** |
| C10-DC | 17.3 | 0.107 |
| log(C16) | 0.228 | 0.075 |
| log(C18) | 0.194 | 0.157 |
| log(C18:2) | 0.226 | **0.020** |
|  |  | |
| **Multivariable** | **log(MLVWT)** | |
|  | **Adjusted coefficient** | **P-value** |
| log(C3) | 0.111 | 0.290 |
| log(C4) | 0.136 | 0.104 |
| C6-DC | 5.00 | **0.004** |
| C8:1 | 0.547 | 0.076 |
| C10-DC | 10.4 | 0.316 |
| log(C16) | 0.124 | 0.323 |
| log(C18) | 0.038 | 0.787 |
| log(C18:2) | 0.120 | 0.240 |

Results from univariable and multivariable linear regression (adjusted for age, sex and body surface area), restricted to subjects without septal reduction therapy. MLVWT, maximum wall thickness.

## Supplemental Figure 1. Overlap between constituent endpoints


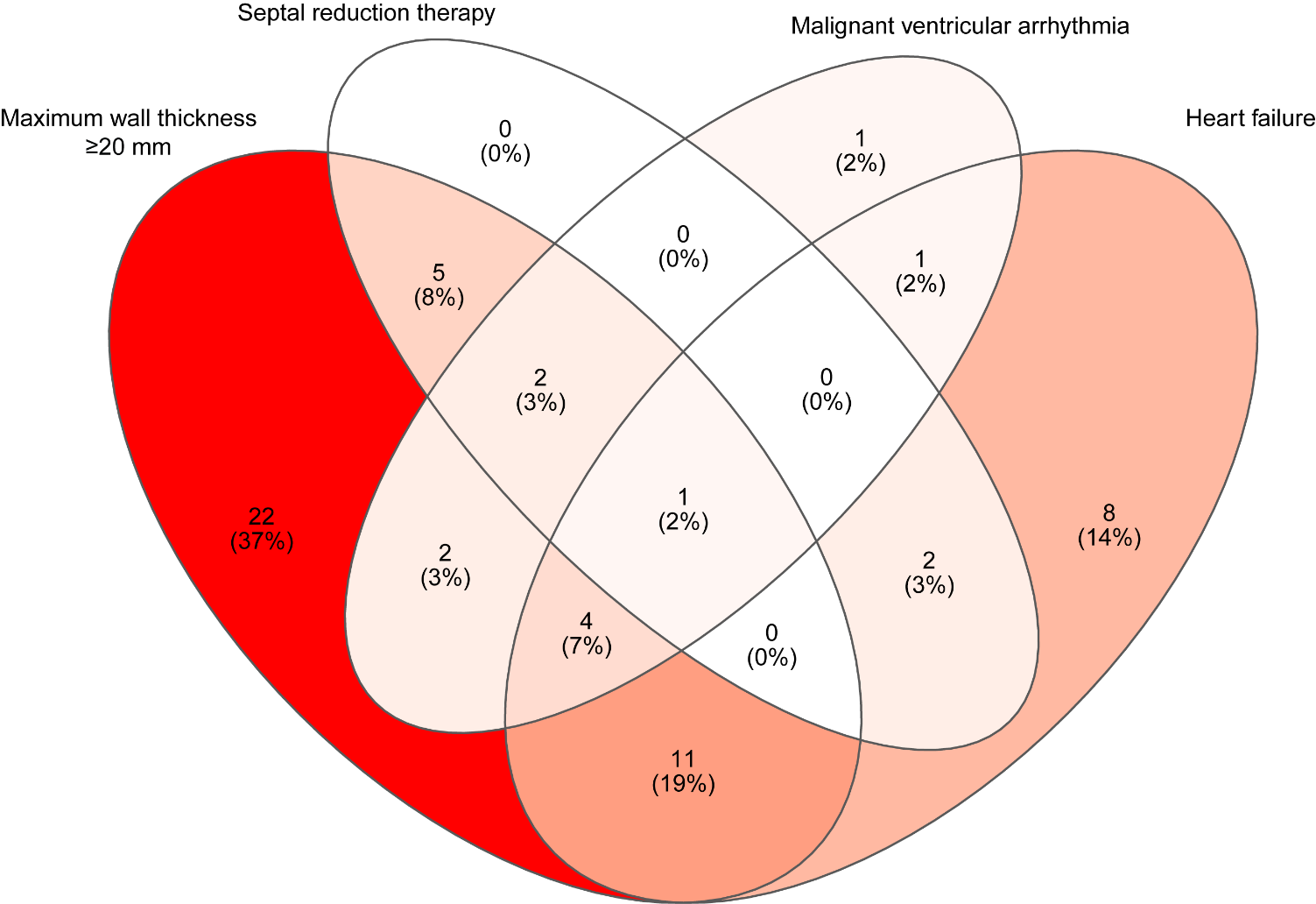


Venn diagram showing the numbers of subjects (%) that fulfilled each constituent endpoint of HCM severity, depicting the overlap between endpoints. Colours are scaled from white to red to reflect the relative number of subjects fulfilling each combination of endpoints.

## Supplemental Figure 2. Correlations between acylcarnitines


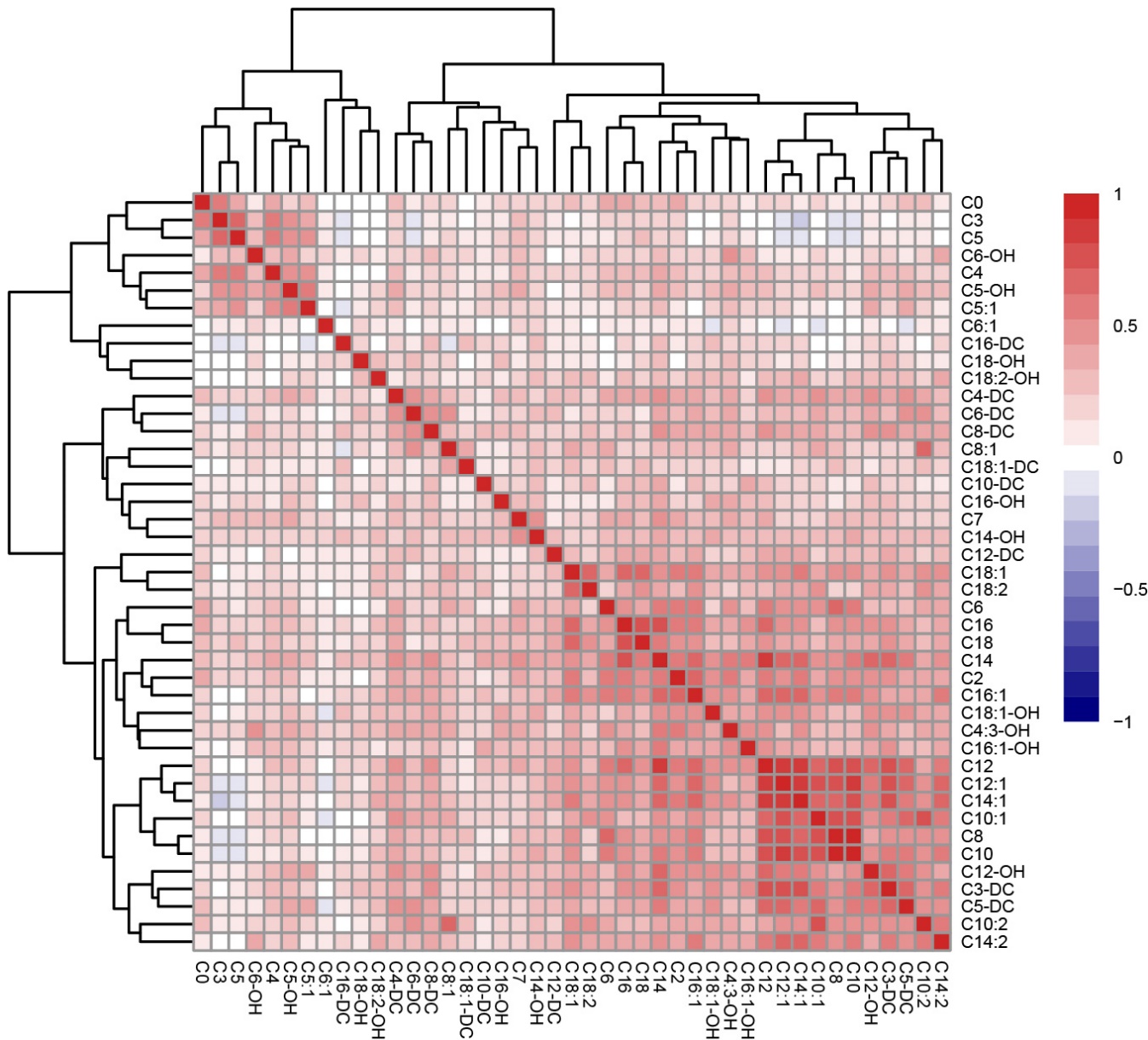


Heatmap of the correlations between acylcarnitines. Correlations were determined using Spearman’s rank correlation coefficient.
